# Continuous Versus Intermittent Nutrition in Pediatric Intensive Care Patients: Protocol for a Randomized Controlled Trial

**DOI:** 10.2196/36229

**Published:** 2022-06-23

**Authors:** Karlien Veldscholte, Arnout B G Cramer, Rogier C J de Jonge, Renate D Eveleens, Koenraad F M Joosten, Sascha C A T Verbruggen

**Affiliations:** 1 Intensive Care Unit Department of Pediatrics and Pediatric Surgery Erasmus MC - Sophia Children's Hospital, University Medical Center Rotterdam Rotterdam Netherlands; 2 Department of Anesthesiology Location Amsterdam Medical Center Amsterdam University Medical Centers Amsterdam Netherlands

**Keywords:** pediatric intensive care unit, PICU, pediatric critical illness, time-restricted feeding, intermittent fasting, feeding intolerance, ketones, circadian rhythm

## Abstract

**Background:**

*Intermittent fasting* is a time-restricted feeding strategy with proven health benefits, which is based on multiple metabolic and endocrine changes, in several patient populations and healthy participants. In the pediatric intensive care unit (PICU), artificial feeding is usually administered 24 hours a day, although solid evidence supporting this practice is lacking. This discards the potential benefits of fasting in this population. We hypothesize that intermittent nutrition with a focus on an overnight feeding interruption (*intermittent fasting*), as compared with 24-hour continuous nutrition, is a feasible and safe strategy, with potential benefits, for critically ill children.

**Objective:**

The aim of the Continuous versus Intermittent Nutrition in Pediatric Intensive Care randomized controlled trial (RCT) is to investigate a strategy of intermittent nutrition with a focus on an overnight feeding interruption period versus 24-hour nutrition during the first 14 days in the PICU.

**Methods:**

The *Continuous versus Intermittent Nutrition in Pediatric Intensive Care* study is an investigator-initiated RCT in a tertiary referral PICU. Critically ill children (term newborn to 18 years), expected to stay in the PICU for ≥48 hours, and dependent on artificial nutrition, are eligible for inclusion. This study will randomize critically ill children (n=140) to a *continuous* versus *intermittent* nutrition strategy. In both groups, similar daily caloric targets will be prescribed. In the *continuous* group (control), nutrition will be administered 24 hours a day, with a maximum interruption period of 2 hours. In the *intermittent* group (intervention), nutrition will be interrupted during an age-dependent overnight fasting period. The study intervention will last until admission day 14, initiation of oral intake, or discharge from the PICU, whichever comes first. The primary outcome is the difference in ketosis between the groups under the condition of noninferiority regarding caloric intake. Secondary outcomes are feeding intolerance; the proportion of severe and resistant hypoglycemic events and severe gastrointestinal complications; and additional observed effects on nutritional intake, circadian rhythm, and clinically relevant outcome measures of the intermittent feeding strategy compared with continuous nutrition.

**Results:**

The study was approved by the Dutch national ethical review board in February 2020. The first patient was enrolled on May 19, 2020. By May 2022, a total of 132 patients had been included in the study. Recruitment of the last patient is expected in Q3 2022.

**Conclusions:**

Although *intermittent fasting* has been proven to have many health benefits in both animal and human studies, the feasibility and safety of this strategy in a PICU setting must be investigated. This RCT will help physicians gain more insight into the feasibility, safety, and potential clinical effects of intermittent feeding with overnight fasting in critically ill children.

**Trial Registration:**

Netherlands Trial Register NL7877; https://trialsearch.who.int/Trial2.aspx?TrialID=NL7877

**International Registered Report Identifier (IRRID):**

DERR1-10.2196/36229

## Introduction

### Nutritional Support for Children in Intensive Care

For a long time, observational studies have suggested that a substantial proportion of critically ill children, most notably infants, develop a pronounced caloric deficit, which has been associated with adverse outcomes [1-4]. This led to recommendations to provide early artificial nutrition via the enteral or parenteral route, targeting to cover at least the calculated or measured resting energy expenditure (REE), compensate for negative protein balance in critical illness, attenuate catabolism, and avoid, possible lethal, complications [5]. To be able to reach caloric targets early in this patient category that is frequently perceived as intolerant to enteral feeding, continuous 24-hour feeding has become a preferable strategy [6].

However, recently, the focus on high macronutrient intake in the acute phase of critical illness has been reconsidered. *The Pediatric Early versus Late Parenteral Nutrition in the Intensive Care Unit (PEPaNIC) randomized controlled trial (RCT)* revealed the beneficial effects of withholding parenteral nutrition (PN) and accepting a macronutrient deficit during the first week of critical illness, such as a decrease in the incidence of new infections, a shortening of the length of stay in the pediatric intensive care unit (PICU) and in the hospital, and a reduction in health care costs [7-9]. This strategy of accepting relative underfeeding of macronutrients in the first week of critical illness has even been shown to be beneficial in patients who are most vulnerable to low nutritional intakes, such as patients who are undernourished and term neonates [10,11]. Withholding PN had no negative effect on participants’ anthropometric measurements during their PICU stay. Moreover, it did not negatively affect their long-term health but actually improved certain parts of their neurocognitive outcome 2 and 4 years later [12,13]. These counterintuitive findings of the beneficial impact of low macronutrient intake during the first week of critical illness were at least partially explained by a fasting response, as observed with increased ketogenesis [14].

### Intermittent Fasting During Pediatric Critical Illness

Owing to the *Pediatric Early versus Late Parenteral Nutrition in the Intensive Care Unit* trial, the potential benefit of macronutrient restriction, or a fasting response, during the first week of critical illness is more recognized. However, knowledge on when safe fasting ends and potentially detrimental effects of starvation start, is lacking. An alternative *fasting mimicking* strategy could be an intermittent feeding pattern, in which a daily fasting response is induced while still providing sufficient amounts of nutrients. *Intermittent fasting* has been widely studied, and the observed health effects of these intermittent feeding strategies are based on changes in metabolic, endocrine, and epigenetic pathways that are also crucial in critical illness [15].

### Aims

Although evenly distributing the daily caloric intake over 24 hours has long been perceived to be more feasible than intermittent feeding in critically ill children, hard evidence for the superiority of continuous feeding over intermittent fasting is lacking [5]. All of the available studies on intermittent feeding in critically ill children examined bolus feeding, and none of those examined the potential of overnight fasting [15]. Currently, no data are available on when the fasting response in critically ill children commences. Furthermore, the severity of illness and nutritional status of the child probably affect the dynamics of a fasting response [15]. Therefore, a study examining the fasting response and potential impact of overnight fasting in critically ill children is warranted. Therefore, the aim of the *Continuous versus Intermittent Nutrition in Pediatric Intensive Care* (ContInNuPIC) RCT is to investigate a strategy of intermittent nutrition with a focus on an overnight feeding interruption period versus 24-hour nutrition during the first 14 days in the PICU.

## Methods

### Ethics Approval and Informed Consent Procedure

The protocol and informed consent forms were approved by the Dutch national ethical review board *Centrale Commissie Mensgebonden Onderzoek* (CCMO; NL72302.000.19). The monitor verifies that the trial will be performed in accordance with the protocol described in the European Medicine Agency’s *Note for guidance on good clinical practice CPMP/ICH/135/95* and the *Declaration of Helsinki*. Eligible patients and, if applicable, their parents or legal guardians, will be informed by one of the members of the research team orally in plain language and in writing. This member of the research team will not be involved in the treatment of the patient. Informed consent will be provided in writing by the parents or legal guardians and confirmed by the child if they are aged ≥12 years. Patients aged ≥16 years will be asked for consent if possible; otherwise, parents or legal guardians will act as legal representatives. For planned admissions, informed consent will be obtained before surgery if possible. For unplanned admissions (or patients with planned admissions whose consent could not be obtained before surgery), informed consent will be obtained within 24 hours after becoming eligible for the study.

### Patients’ Eligibility

#### Inclusion Criteria

Upon admission to the PICU, all the children will be screened for eligibility for inclusion in the ContInNuPIC clinical study. All patients identified by the research team will be logged.

Critically ill children, term newborn to aged 18 years, who are likely to stay in the PICU for >48 hours and to be dependent on artificial nutrition, are eligible for inclusion.

Patients will be considered critically ill if they meet at least one of the following criteria:

- Respiratory support (excluding low flow nasal cannula)
- Hemodynamic support (pharmacological or mechanical)
- Continuous renal replacement therapy on account of acute renal failure

Patients already on home respiratory support will be considered critically ill only if they have significantly deteriorated compared with normal functioning; that is, need for an increase in the level of support (eg, higher pressure rates, higher fraction of inspired oxygen, or need for hemodynamic support).

#### Exclusion Criteria

Patients fulfilling ≥1 of the following criteria will be excluded:

- Preterm neonates (<37 weeks postmenstrual age [PMA] upon admission to the PICU)
- *Do not resuscitate* code at the time of PICU admission
- Expected death within 24 hours
- Readmission to the PICU >48 hours after already having been included in the ContInNuPIC trial
- Transfer from another PICU or neonatal intensive care unit (ICU) after a stay of ≥3 days or having received artificial nutrition (any PN or enteral nutrition [EN] with a caloric intake >10% of predicted REE per day)
- Ketoacidotic or hyperosmolar coma
- Metabolic diseases requiring a specific diet or with a contraindication to (intermittent) feeding
- Short bowel syndrome or other conditions requiring PN before admission
- Participation in another RCT in the PICU with an intervention that might influence the clinical outcome

### Data Collection at Study Entry

At baseline, data on demographic (age, sex, race, ethnicity, and preadmission body weight, and height) and clinical characteristics of the patients will be obtained. For all patients, risk of mortality scores (*Pediatric Index of Mortality score*) [16], disease severity scores (*Pediatric Logistic Organ Dysfunction score*) [17], and the nutritional risk score (*Screening Tool for Risk on Nutritional status and Growth score*) [18] will be calculated, and the *Risk-Adjustment in Congenital Heart Surgery* classification [19] for patients of cardiac surgery will be recorded. In addition, comorbidities, such as the presence of a congenital disease or syndrome, gestational age at birth, presence or history of cancer, diabetes mellitus, kidney disease, liver disease, chronic heart disease, home ventilatory support, and sepsis upon admission, will be noted. In addition, for all patients, baseline results of routine clinical chemistry will be recorded.

### Randomization Procedure

Randomization of participants to *continuous* or *intermittent* nutrition groups will be performed using the ALEA randomization tool (ALEA Clinical, FormsVision), a dedicated computerized system accessible 24 hours a day and 7 days a week. The computer algorithm allocates every consecutive eligible patient to 1 of the 2 treatment arms in a one-to-one allocation ratio using permuted blocks of 10. Patients will be stratified into 3 age groups: neonates (≤44 weeks PMA), infants (<1 year), and children (≥1 year).

### Blinding

It was considered not feasible to blind treating physicians and patients for the allocated treatment during the time window of the randomized intervention. All outcome assessors and investigators not directly involved in patient care, such as statisticians and laboratory personnel, will be fully blinded to the treatment allocation.

### Protocol Adherence

The medical and nursing staff of the PICU were informed and trained extensively during regular meetings before the start of the trial and were familiarized with the protocol. To optimize protocol compliance, reminders for study interventions and measurements will be incorporated into the electronic patient data management system. Moreover, the research team will provide a daily follow-up of all included patients.

### Nutritional Support

#### Caloric Intake

**Figure 1:**
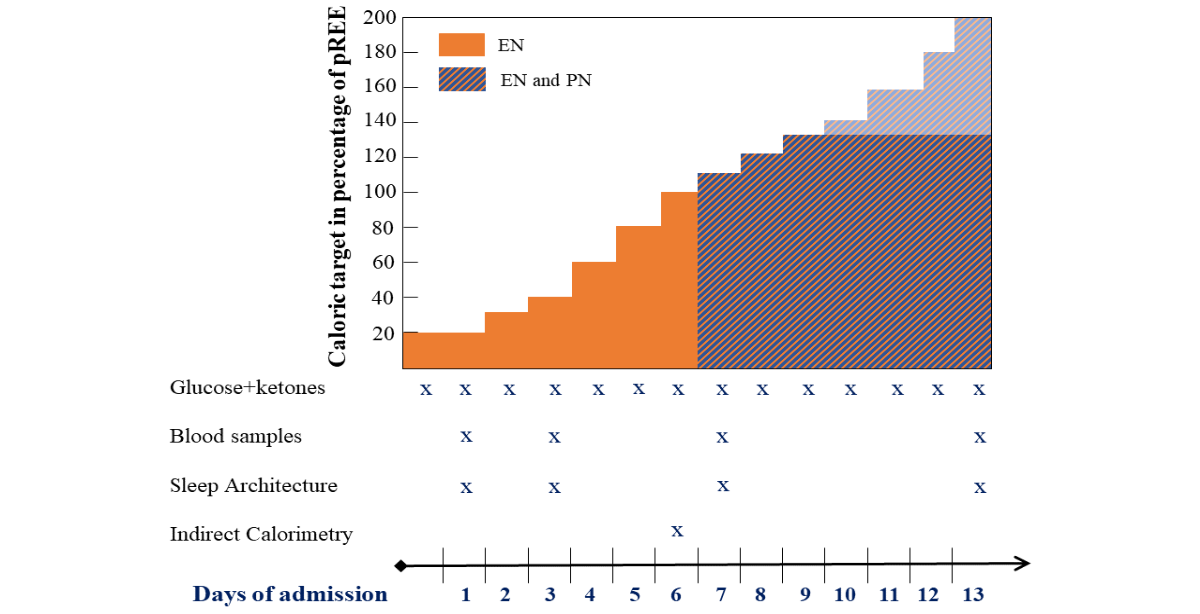
Daily caloric target and timeline of primary study measurements. For details, see text. EN: enteral nutrition; PN: parenteral nutrition; REE: resting energy expenditure.

During the study, nutritional intake will be provided to both allocation groups according to the current pediatric critical care nutrition guidelines [5,20,21]. In all patients from both study arms, EN will be started as soon as possible (<24 hours), provided that they are hemodynamically stable and without formal contraindications. EN will be administered through a gastric or postpyloric tube and gradually increased using a stepwise protocol (Figure 1), guided by the clinical judgment of the responsible clinician. Caloric goals will be calculated upon admission according to the body weight–based or body weight and length–based Schofield equation for the estimation of REE [22]. In line with the international nutritional guidelines for critically ill children, caloric intake is targeted to reach 1×REE at the end of the first week [5]. After 1 week, caloric target intake increases further to a maximum caloric intake of 1.3×REE for older children and adolescents and up to 2×REE for neonates and infants [20]. The research team will provide nutritional advice based on the standard protocol on a daily basis. The responsible clinician will decide the actual amount of nutrition prescribed based on the protocol, the nutritional intake the day before, and the tolerance of the administered nutrition. If EN cannot be started or increased according to the protocol, the reasons will be recorded in the database.

#### EN Support

In neonates and infants, breast milk, the patient’s standard milk formula, or a protein energy–dense formula will be used. For patients aged >1 week, breast milk will be enriched with breast milk fortifiers, and if a protein energy–dense formula is indicated, other fortifiers such as triglycerides can be added as well. Older children usually receive a standard enteral feeding formula or a protein energy–dense formula. A protein energy–dense formula will be used in case the patient is subjected to strict fluid restrictions or is unable to reach caloric targets. Hydrolyzed or semielemental formulas will be used when patients are allergic to or do not tolerate regular formulas (Table 1) [23].

**Table 1:** Stepwise approach for nutritional therapy in the pediatric intensive care unit^a^.

Step	Nutritional therapy	Consideration
Step 1	Infants: standard polymeric formula or breast milk Children: standard polymeric formula	May result in nutritional deficits because of the lower energy and protein content of these formulas and breast milk
Step 2	Polymeric protein: energy-enriched formula	Higher energy and protein content may overcome nutritional deficits, especially in patients with fluid restriction
Step 3	Semielemental protein: energy-enriched formula	Absorption, tolerance, and use of proteins and fats may be altered, and semielemental feeds are considered as an alternative
Step 4	If insufficient EN^b^ (<80%) or no EN is possible >1 week after admission: start PN^c^	Especially in children with intestinal failure; appropriate growth and normal body composition difficult to achieve and risk of associated liver disease

^a^Source: Joosten et al [23].

^b^EN: enteral nutrition.

^c^PN: parenteral nutrition.

#### PN Support

During the first week, only EN without supplemental PN will be provided to conform with current guidelines [5,20]. Beyond day 7, if EN is insufficient (<80% of the target intake), parenteral macronutrients will be additionally provided through PN until EN reaches >80% of the target intake. If standard PN formulas are not suitable or are contraindicated for the patient, a tailor-made PN formula can be ordered by the dietician. To reduce the risk of refeeding syndrome on the first day of PN, half the target amount of PN will be provided [24].

### Intervention Group: Intermittent Nutrition

**Figure 2:**
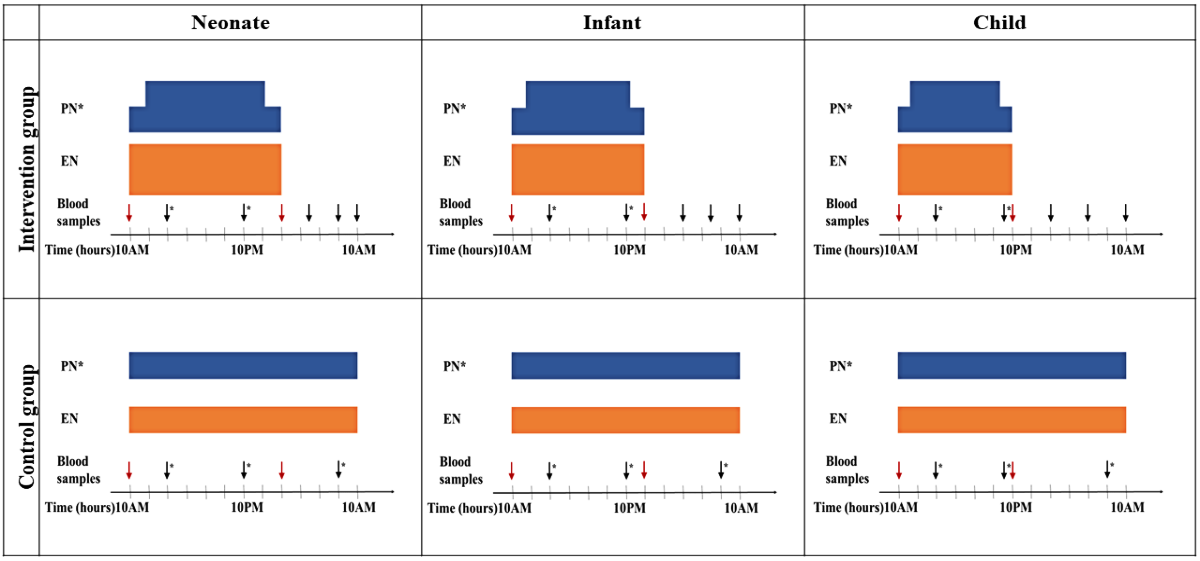
Randomization and age group–specific feeding schedules and measurements. Glucose and ketone level measurements are indicated with black arrows; during the fasting interval these measurements are performed at set time points (black arrows without asterisk); during feeding these measurements are performed regularly, but not at specific time points (black arrows with asterisk). Blood samples for metabolism analyses are at set time points in both fasted and fed state and are indicated with red arrows. *Parenteral nutrition is only supplied in the second week of pediatric intensive care unit (PICU) admission, when enteral nutrition is insufficient. For details, see text. EN: enteral nutrition; PN: parenteral nutrition.

For patients randomized to the *intermittent* nutrition group (intervention group), all nutritional support will be provided during the day and withheld during the overnight feeding interruption period. The overnight feeding interruption period will be designed in an age-dependent manner. The feeding interruption period will be from 2 AM to 10 AM in neonates (≤44 weeks PMA), from 12 AM until 10 AM in infants (<1 year), and from 10 PM to 10 AM in children (≥1 year), as shown in Figure 2. The daily planned nutritional intake will be provided during the condensed feeding period over the day. The starting time of nutritional intake is set to 10 AM to facilitate procedures that require fasting to be performed in the morning. PN (beyond day 7, if necessary) will be provided during the daily age-dependent feeding periods and tapered over 1 hour during the starting and stopping of PN to reduce the risk of swift metabolic shifts (Figure 2) [25,26].

### Control Group: Continuous Nutrition

Patients randomized to the *continuous* nutrition strategy (control group) will receive nutrition according to current management for 24 hours a day, with a maximum planned interruption period of 2 hours (Figure 2). Planned feeding interruptions exclude interruptions because of clinical ICU care (eg, interventions such as intubation and surgical procedures). Feeding interruptions, timing, and reason will be recorded in the database. PN (beyond day 7, if necessary) will be provided continuously for 24 hours a day without interruption (Figure 2).

### Medication and Supplementation

In all patients, medication will be provided in sodium chloride–based intravenous fluids instead of glucose-based intravenous fluids unless contraindicated. Medication and fluids can also be administered during the overnight feeding interruption period. If EN is insufficient (<80% target intake) during the first week, parenteral trace elements, minerals, and vitamins will be administered daily to all patients in both treatment groups similarly [27]. Intravenous micronutrient substitution will be stopped when patients receive at least 80% of their caloric needs via the enteral route.

### Blood Glucose Management

Upon request of the national ethical review board (CCMO) on account of safety concerns, for patients in the intervention group, a glucose intake infusion will be provided during the overnight feeding interruption period (1.0 mg/kg/minute in neonates and 0.5 mg/kg/minute in infants) to reduce the risk of hypoglycemia. In children aged >1 year, no baseline glucose infusion during the overnight feeding interruption period will be provided unless clinically indicated. Glucose level measurements will be performed at least once daily during the feeding period, regularly during fasting intervals and when clinically indicated. Blood glucose management will be performed according to the local protocol. In short, blood glucose levels will be targeted from 4.0 to 7.8 mmol/L in all age groups. In patients with hyperglycemia (glucose >10 mmol/L) in 2 measurements (with 1 hour between measurements), the glucose intake will be first reduced, and if this is not sufficient, a continuous insulin infusion will be started using a stepwise nurse-driven glucose control protocol. If the blood glucose levels are <2.6 mmol/L, a bolus of 1 mL/kg glucose 10% will be provided and the baseline glucose infusion will be increased until the blood glucose level is >4.0 mmol/L. The glucose levels will be measured again within 30 minutes and 1 hour of treatment. In patients randomized in the *intermittent* group, if insulin is provided, this will be stopped 1 hour before the start of the overnight feeding interruption period.

### Other Procedures and Guidelines

No other medical treatments were prescribed in the study protocol. The patients will be weaned from the ventilator and from hemodynamic support according to standardized guidelines. As the timing of PICU discharge to a regular ward may be affected by the availability of beds in regular wards, which could induce bias, we will analyze the *time to discharge from PICU* as the *time to being ready for discharge from PICU*. A patient is considered *ready for discharge* as soon as all clinical conditions for PICU discharge have been fulfilled (no longer in need of, or at risk of, vital organ support).

### Per-Protocol Stop of Study

A switch to oral intake will be made as soon as it is deemed safe by the clinician responsible for the patient.

The study intervention will last until admission day 14, until the child could be fed by mouth (oral feeding), or until discharge from the PICU, whichever comes first. After day 14, the intervention will stop, and nutritional support will be provided according to standing orders when oral intake remains insufficient.

### Discontinuation of the Study Intervention

We defined a stopping rule (no-go) for individual patients to be withdrawn from the study who develop the following:

- Hypoglycemia (<2.6 mmol/L) with clinical symptoms (eg, pallor, transpiration, irritability, lethargy, loss of consciousness, and convulsions) or severe hypoglycemia (<2.2 mmol/L) *and* who are resistant to parenteral glucose administration; *resistance* is defined as no elevation of glucose within 1 hour after parenteral glucose bolus (1 mL/kg glucose 10%) administration and elevation (double) of standard glucose infusion via the standard local protocol
- Acute, nonocclusive mesenteric ischemia (ie, intestinal perforation and necrotizing enterocolitis) not attributed to an anatomical substrate (eg, volvulus)

### Handling of Readmissions to the PICU

Patients participating in the study who are readmitted to the PICU within 48 hours of discharge and who are still within the 14-day time window of the initial randomization will continue to receive the nutrition strategy they were randomly assigned to during their initial PICU admission. Patients readmitted >48 hours after PICU discharge after participation in the ContInNuPIC study will not be eligible for reinclusion and will be fed at the discretion of the attending physician.

### Study Blood Samples

For all patients, daily plasma glucose levels will be measured and analyzed at the bedside using a blood gas analyzer (ABL90 FLEX PLUS, Radiometer) or point of care meter (Accu-Chek Inform II, Roche Diabetes Care), and daily ketone levels (3-β-hydroxybutyrate) will be measured using the StatStrip Glucose/Ketone meter (Nova Biomedical). In the *intermittent* feeding group, glucose and ketone levels will be measured more frequently during the feeding interruption period (Figure 2).

Additional blood samples for other analyses of metabolism (eg, glucagon, insulin, and free fatty acids) and circadian rhythm (eg, gene expression and cortisol and melatonin) will be taken upon ICU admission and on days 3, 7, and 14 (Figure 2). The blood samples for metabolism will be collected using serum-separating tubes in both fasted (before 10 AM) and fed states (before 10 PM, 12 AM, or 2 AM, depending on the age group) for the intervention group or at similar time points for the continuous group. The blood samples for circadian rhythm will be collected in EDTA tubes every 4 hours during a 24-hour period (a total of 7 samples per 24 hours). The blood samples for gene expression will be collected in *PAXgene* RNA tubes (PreAnalytiX) once daily on the selected days at 10 AM. These additional blood samples will be taken from lines placed for clinical purposes or in combination with draws requested for clinical purposes. Owing to ethical considerations, the number of blood samples will be calculated using the body weight of the individual patient to ensure a maximum of 5% of blood volume for the entire study.

After collection, the blood samples will be immediately stored in a refrigerator. Within 24 hours after collection, the blood samples from serum-separating tubes and EDTA tubes will be centrifuged at 20 °C, and serum or plasma will then be separated and stored at −80 °C for future measurements. The *PAXgene* tubes will not be centrifuged and will be first stored at −20 °C for 1 day before storage at −80 °C to prevent breakage of the tubes. In addition, plasma phosphate, magnesium, and potassium levels will be determined in residuals of clinical plasma samples by the clinical chemical laboratory on days 0 to 4, 7 to 10, and 14.

### Circadian Rhythm Measurements

The circadian rhythm is expected to be affected by the feeding strategy. Electroencephalography, electrooculography, and electromyography will be recorded for 24 hours on admission, day 3, day 7, and day 14 (with a margin of 1 day) or before discharge if the discharge is before day 14. Multichannel registration will be performed using a standard device (Brain RT, OSG or Morpheus, Micromed SpA). Melatonin and cortisol will be measured in the study blood samples, as melatonin and cortisol both have a clear relationship with circadian rhythms [28,29]. The blood samples used for gene expression analysis may be used to determine the internal time of peripheral blood mononuclear cells [30]. The difference between this internal time and the time of blood draw is a measure of circadian disturbances. In addition to the blood samples, salivary melatonin and cortisol levels will be measured to evaluate the possibility of measuring the circadian rhythm in saliva [31]. As ambient light and sound may affect and thus confound circadian rhythm [32,33], quantitative light and sound measurements will be taken at the bedside during ICU stay. All routinely monitored vital signs (including heart rate, respiratory rate, oxygen saturation, perfusion index, body temperature, central venous pressure, end-tidal carbon dioxide, arterial blood pressure, oxygen saturation, and electrocardiogram waveforms) will be stored at 1 Hz to 200 Hz intervals for further analysis.

### Data Handling and Record-Keeping

Data will be collected electronically in a pseudonymized electronic case record form (eCRF) with an audit trail unambiguously linked to the source file using the *OpenClinica* clinical data management system (OpenClinica Community; version 3.12.2; OpenClinica LLC). Every day, the types and amounts of EN and PN delivered will be registered in the eCRF. Interruptions in EN delivery and gastrointestinal intolerance will be registered daily. The duration (in minutes) and the cause of interruption of EN delivery will be recorded. The data will be manually transferred into and checked for accuracy in the eCRF by the research team. Extensive range and consistency checks will be performed by the monitor of the study. All systemically administered medications, analyses of routine clinical chemistry, and whole blood glucose levels during the stay in PICU will be registered in the electronic patient file to be retrieved after completion of the study. The vital status at 30 days (and at later follow-up times) will be recorded for all patients in consultation with the National Personal Records Database. When this information is not available, the vital status will be checked through the hospital information system or regional network of pediatricians and general practitioners. All original records, such as consent forms, case report forms, and relevant correspondence, will be archived according to national regulations.

### Outcome Measures

#### Primary End Points

The primary outcome of this study will be the feasibility of a daily feeding and fasting cycle (*intermittent feeding* strategy) compared with a continuous 24 hours per day feeding strategy in critically ill children. The primary outcome is defined as the difference in ketosis (3-β-hydroxybutyrate levels) between the groups under the condition of noninferiority regarding caloric intake. The amount of nutritional intake is defined as the daily caloric intake as a percentage of the estimated REE based on the body weight–based or body weight and length–based Schofield equation.

#### Secondary End Points

Secondary end points will comprise outcomes regarding the safety, feasibility, and efficacy of the intervention. The primary safety outcome is defined as the difference in the incidence of severe and resistant hypoglycemic events and severe gastrointestinal complications between the groups. For definitions of severe and resistant hypoglycemia and severe gastrointestinal complications, see the *Discontinuation of the Study Intervention* section*.*

The secondary safety outcomes comprise the following:

- Overall blood glucose control (hypoglycemic incidents and hyperglycemic incidents)
- Complications of nutritional support
- Hyperlactatemia during the feeding interval

As patients with a feeding and fasting cycle in the *intermittent* group may be considered at increased risk for hypoglycemia during the fasting interval, we will also report hypoglycemic incidents (glucose <2.2 mmol/L) during the time window of the randomized intervention for both groups as the number of nights with at least one episode of hypoglycemia divided by the total number of days of the intervention. As patients in the *intermittent* group may be considered at increased risk for hyperglycemia during the condensed feeding period, we will report the hyperglycemic incidents (glucose >10 mmol/L) during the time window of the randomized intervention for both groups as the number of days with at least one episode of hyperglycemia divided by the total days of the intervention.

Possible complications of nutritional support are shown in Textbox 1.

Hyperlactatemia is defined as an increase in arterial lactate levels above 2 mmol/L during the feeding interval.

Other secondary outcomes will be feeding intolerance and metabolic (eg, course of ketones, lactate, and free fatty acids) and endocrine responses (eg, insulin and glucagon). Enteral feeding intolerance is defined according to the definition by Eveleens et al [34] (Multimedia Appendix 1). Furthermore, we will evaluate any observed effects of the intermittent feeding strategy compared with continuous nutrition on nutritional intake, circadian rhythm, and clinically relevant outcome measures.

The effects on nutritional intake and circadian rhythm that will be investigated are presented in Textbox 2.

Clinically relevant outcome measures will be collected until 30 days after admission and are presented in Textbox 3.

**Possible complications of nutritional support.**

**Complications possibly related to feeding tubes:**

- Complicated insertion (eg, nasal bleeding)
- Mechanical complications (feeding tube displacement and obstruction)

**Complications possibly related to parenteral feeding:**

- Mechanical complications (occlusion and dislodging of central venous catheters)
- Clinical complications (pneumothorax, hemothorax, arterial puncture, and central line replacement because of the suspicion of catheter-related bloodstream infections)

**Possible effects on nutritional intake and circadian rhythm.**

**Nutritional intake**

- The proportion of patients receiving at least a caloric intake of 67% of predicted resting energy expenditure (pREE) before or at the end of the first week and the proportion of patients receiving at least a caloric intake of 100% of pREE before or at the end of the first week
- The proportion of days caloric targets are reached divided by the total days in the study per patient
- Median caloric intake as a percentage of pREE
- The proportion of patients requiring supplemental parental nutrition beyond day 7
- The time to oral intake

**Circadian rhythm**

- Cortisol and melatonin patterns in blood and in saliva
- Circadian rhythm in gene expression
- Sleep architecture (quantity and quality)
- Circadian rhythm in vital signs

**Clinically relevant outcome measures.**

**Clinically relevant outcome measures**

- Time to final (live) weaning from mechanical respiratory support and duration of mechanical ventilation
- Time to final (live) weaning from pharmacological or mechanical hemodynamic support and duration of such support
- Time to (live) discharge from the pediatric intensive care unit (PICU) and duration of PICU stay
- Time to (live) discharge from the hospital and duration of hospital stay for both the index hospitalization and total hospitalization, including stay in the referred hospital
- Newly acquired infections, which are defined as a microbiologically or laboratory-confirmed infection in combination with the start of treatment or clinical suspicion of infection, as assessed by the clinician responsible for the patient
- Mortality during the time window of the randomized intervention during PICU stay, during hospital stay, and after 30 days
- Acute kidney failure: Patients in need of renal replacement therapy (RRT) during PICU stay and the duration of RRT (for patients requiring RRT); Patients with acute kidney injury according to the *Pediatric Risk, Injury, Failure, Loss, End Stage Renal Disease* criteria [35]
- Patients with newly acquired liver failure according to the *Pediatric Acute Liver Failure* criteria or with a clinical diagnosis of acute liver failure [36]

#### Other Outcomes

Further metabolomic, endocrine, and inflammatory measurements on stored samples in the context of mechanistic analyses are planned. Moreover, these samples may be used for pharmacokinetics, pharmacodynamics, and autophagy analyses. In addition, we will evaluate parental stress (*Parental Stress Score*–PICU) [37] and the overall workload among (PICU) nurses (National Aeronautics and Space Administration Task Load Index) [38]. The detailed protocols and methods for the statistical analyses for these outcomes will be reported separately. Finally, a follow-up study after enrollment in the ContInNuPIC (concerning, eg, neurocognitive and physical function) will be planned as well; however, the details are beyond the scope of this study protocol.

### Trial Organization

The sponsor (Erasmus MC, Rotterdam, The Netherlands) provides direct access to the eCRF, the source data, and the trial master file for monitoring and regulatory inspection. The sponsor has appointed 1 monitor. Monitoring will be performed and reported according to the sponsor’s standard operating procedures. The clinical research team guarantees a daily follow-up of patient screening and inclusion, availability of requested clinical data in the clinical patient files, and protocol compliance. Noncompliance to the protocol and other questions or problems will be discussed with the principal investigator and reported to the study monitor. Regular meetings will be organized between the principal investigator and clinical research team to discuss the daily progression of the ContInNuPIC trial. Serious adverse events occurring during the intervention will be directly reported to the sponsor and the accredited ethical commission (CCMO) and registered in the database.

### Statistical Analysis

#### General Rules

A CONSORT (Consolidated Standards of Reporting Trials) flow diagram will be reported [39]. All analyses will be primarily performed according to an intention-to-treat analysis. To perform additional per-protocol analysis, we anticipated that up to 30% of the patients will not be able to receive artificial nutrition during their admission to the PICU.

Categorical variables will be summarized as counts and frequencies and analyzed using the chi-square test or Fisher exact test. Continuous variables will be summarized using either mean and SD or median and IQR, depending on the distribution of the variables.

All analyses will be performed primarily for the complete group. In secondary analyses, analyses can be stratified according to different age groups (neonates, infants, and children). Adjustment for baseline risk factors, including the diagnostic group, age group, severity of illness, and severity of nutritional risk, will only be used for secondary analyses. Multiple imputation will only be used in secondary analyses to deal with missing data for confounders. For all end points, differences will be considered statistically significant when the 2-sided *P* value is <.05 or when the 1-sided *P* value is <.025 in the case of noninferiority tests, without correcting for multiple testing.

#### Primary Outcomes

To assess a significant difference in ketosis between the 2 arms, a 2-tailed *t* test will be performed on the patient means of the highest ketone levels in each fasting period (or similar time point in the control group). A linear mixed model will be used to assess the noninferiority of caloric intake (repeatedly measured over time) in the intervention group, with a noninferiority margin of 33%.

#### Secondary Outcomes

The statistical analysis plan for secondary outcomes will be published separately. In short, the safety of the intervention will be assessed by comparing the incidence of both severe and resistant hypoglycemic events and severe gastrointestinal complications using the Cox proportional hazard analysis. Feeding intolerance will be analyzed using a mixed effects logistic model. Moreover, mixed models will be used for the assessment of other longitudinal data such as analyses of the course of ketones and other metabolic markers. Kaplan-Meier plots will be used to document time-to-event effects, and the time-to-event effect size will be estimated using the Cox proportional hazard analysis. All time-to-event analyses will be performed on data censored at 30 days. As death is a competing risk for the duration of care outcomes, nonsurvivors will be censored beyond the longest duration of such care required for survivors.

### Sample Size Calculation

To provide a careful estimate of the number of patients required to answer whether the intervention is feasible, we based the sample size on a (1) a fasting response with ketogenesis and (2) sufficient nutritional intake.

We calculated the sample size to detect a fasting response with ketogenesis. In adults who are critically ill (n=70), 12 hours of fasting increased ketones compared with full feeding periods (ketones +0.47, SD 0.07 mmol/L; *P*<.001) [40]. In a study of healthy children, ketones increased from 0.08 mmol/L in the fed state to 0.34 mmol/L (90% CI 0.02-1.78 mmol/L) after 15 hours of fasting [41]. Using these data, we assumed baseline ketones of 0.10 mmol/L (fed state) and 0.20 mmol/L in the fasted state, with an SD of 0.16 mmol/L. To perform additional per-protocol analysis, we increased the sample size by 30%. Ultimately, the sample size of 140 should be able to detect, with at least 90% power (2-tailed power) and 95% certainty, the assumed increase in mean ketone levels of 0.10 mmol/L. Regarding nutritional intake, we considered a reduction in cumulative (caloric) intake, corrected for PICU length of stay, of >33% in the intervention group as clinically relevant based on the currently available literature [2]. The sample size (N=140, 70 per arm) is expected to be able to detect, with at least 80% power (1-tailed power) and 80% certainty, a reduction of 33% in cumulative caloric intake.

## Results

This study was approved by the Dutch national ethical review board (CCMO) in February 2020. The study was initiated on May 11, 2020, and the first patient was enrolled on May 19, 2020. As of May 12, 2022, 132 patients have been included in the ContInNuPIC trial. Recruitment of the last patient is expected in Q3 2022.

## Discussion

### Hypothesis

Although solid evidence for the superiority of continuous feeding over intermittent feeding is lacking, almost all critically ill children worldwide are fed 24 hours a day. This is assumed to result in an improved nutritional intake with fewer gastrointestinal and metabolic complications. However, this approach conceals the fact that a fasting response might be beneficial for the convalescence of critical illness.

We hypothesize that in critically ill children, intermittent feeding with an overnight fasting period will lead to increased ketogenesis without lowering the daily total nutritional intake. Moreover, we hypothesize that this overnight fasting strategy will not affect feeding intolerance, glycemic control, or safety. This hypothesis is currently being tested in this study in a large tertiary referral PICU (Erasmus MC Sophia Children’s Hospital, Rotterdam, the Netherlands).

### The Potential of an Intermittent Feeding Strategy in Pediatric Critical Illness

An important feature of the intermittent feeding strategy that is hypothesized to contribute to the beneficial effects is the transition to ketone body metabolism [42]. This ketone body metabolism contributes to the conservation of brain function and stimulation of several cellular pathways involved in stress resistance, neuroplasticity, and mitochondrial biogenesis [15]. The activation of autophagy, stimulated by the fasting state [43,44], is hypothesized to exert beneficial effects as well, as this is a process that is crucial for cellular function and integrity and recycling of macronutrients and metabolites [45-47]. Moreover, if the fasting period is implemented during the night, it might help preserve the circadian rhythm, as nutrient intake, or the fasting response, is the most important entrainment signal for the peripheral clock system [48,49]. This preservation of the circadian rhythm might be crucial in the treatment of critically ill patients, as the circadian rhythm is believed to be often disrupted in patients who are critically ill, and this is associated with impaired outcomes [50-54]. The preservation of circadian rhythm is hypothesized to improve sleep and metabolism, and it might even exert beneficial effects on tissue repair, immune response, and delirium risk [15]. A possible problem with implementing an overnight fast is the higher nutritional load during the daytime that is needed to reach the same caloric goals. However, only feeding during the daytime and thus delivering nutrition at a time when the body is attuned to food processing and nutrient uptake, might actually improve the feeding tolerance and glycemic control of patients who are critically ill [15].

In addition to the hypothesized beneficial effects because of the transition to a fasting response and preservation of the circadian rhythm, *intermittent fasting* might also have other beneficial effects. Reperfusion injury, muscle weakness, and immune response are believed to benefit from *intermittent fasting* as well [15]. Thus, *intermittent fasting* might be a favorable treatment option for critically ill children.

### Strengths and Limitations

An important strength of this study is that the data collection is extensive, which will allow us to carefully correct for possible confounders in the analysis and further investigate the possible underlying beneficial mechanisms of intermittent feeding. Moreover, the studied patient population is very heterogeneous, which makes the findings generalizable to the general PICU population.

However, this study has some limitations that should be addressed. One of the limitations is the relatively small sample size, which does not allow for the drawing of conclusions regarding the effect on clinical outcomes. If this study shows that an intermittent feeding strategy with overnight fasting is safe and feasible, a larger RCT will be necessary to investigate its impact on clinical outcomes. Another limitation of this study is that some of the secondary outcome measures will not be performed in all patients, as some measurements (such as circadian rhythm measurements) are not suitable for patients who are very critically ill.

### Conclusions

An intermittent feeding strategy with an overnight fasting period would potentially be capable of providing a fasting response while still providing sufficient nutritional intake, thereby improving clinical outcomes. To our knowledge, this is the first study to investigate such an intermittent feeding strategy in critically ill children. This RCT will help PICU physicians gain more insight into the possibility of omitting nutritional support during the night for critically ill children.

## Appendix

Multimedia Appendix 1 Definition for enteral feeding intolerance in critically ill children in whom enteral nutrition is indicated and attempted; registered over a 24-hour period.

## Abbreviations

CCMO: Centrale Commissie Mensgebonden Onderzoek
CONSORT: Consolidated Standards of Reporting Trials
ContInNuPIC: Continuous versus Intermittent Nutrition in Pediatric Intensive Care
eCRF: electronic case record form
EN: enteral nutrition
ICU: intensive care unit
PICU: pediatric intensive care unit
PMA: postmenstrual age
PN: parenteral nutrition
RCT: randomized controlled trial
REE: resting energy expenditure
