# Supplementary material for: Continuous Versus Intermittent Nutrition in Pediatric Intensive Care Patients: Protocol for a Randomized Controlled Trial
Source: JMIR Res Protoc. 2022 Jun 23;11(6):e36229. doi: 10.2196/36229 (PMC9264130; doi:10.2196/36229)
Supplement: Multimedia Appendix 1 [file resprot_v11i6e36229_app1.docx]

**Supplemental Table 1**. Definition for enteral feeding intolerance in critically ill children in whom EN is indicated and attempted; registered over a 24h period

| **1) Insufficient enteral intake:** | | Defined as enteral intake < two-third of prescribed daily target *or* |
| --- | --- | --- |
|  | | EN is withheld for ≥ 48 hours *or* |
|  | | EN is not increased for ≥ 48 hours |
|  | | (Excluding interruptions due to procedures) |
| **AND** | | |
| **2) Presence of at least one of the following criteria:** | | |
| a) GI-symptoms | |  |
|  | 1. Large GRV | Defined as ≥ 50% of the EN delivered in the last 4 hours |
|  | 1. Presence of vomiting | Defined as ≥ 2 times with gastric content in 24h period |
|  | 1. Presence of diarrhea | Defined as ≥ 4 times loose stool with negative fluid balance in 24h period |
| b) Severe GI-symptoms with concern for intestinal ischemia | | - Abdominal distention  - Abdominal pain  - Melena  - Hematochezia |

Abbreviations: EN: enteral nutrition; GI: gastro-intestinal; GRV: gastric residual volume

Critically ill children must both fulfil the first and second criteria to be classified as feeding intolerant according to this definition.
